# Leveraging data, technology, and policy to address disparities for persons living with Alzheimer's disease and Alzheimer's disease related dementias

**DOI:** 10.1002/alz.70186

**Published:** 2025-04-29

**Authors:** Chanee D. Fabius, Jie Chen, Norma B. Coe, Emmanuel F. Drabo, Shekinah Fashaw‐Walters, Maricruz Rivera‐Hernandez, Tina Sadarangani

**Affiliations:** ^1^ Department of Health Policy and Management Johns Hopkins Bloomberg School of Public Health Baltimore Maryland USA; ^2^ Department of Health Policy and Management School of Public Health, University of Maryland at College Park College Park Maryland USA; ^3^ Department of Medical Ethics and Health Policy, Perelman School of Medicine, University of Pennsylvania Philadelphia Pennsylvania USA; ^4^ Department of Health Services, Policy and Practice Brown University School of Public Health Providence Rhode Island USA; ^5^ Roy Meyers College of Nursing New York University New York New York USA

**Keywords:** data accessibility, disparities, health information technology, insurance, long‐term services and supports, payment policy

## Abstract

Addressing disparities related to Alzheimer's disease and Alzheimer's disease related dementias (AD/ADRD) is a priority for policymakers, practitioners, and researchers. In this perspective, we highlight important gaps and opportunities presented during *Session 4: Disparities in Health Care Access, Utilization, and Quality*, of the *2023 National Research Summit on Care, Services, and Supports for Persons Living with Dementia and Their Care Partners/Caregivers*. We call attention to three areas: (1) increased data availability and linkages across local, state, and federal levels; (2) health information technology use and related care access, quality, and costs; and (3) diverse health insurance models used to enable access to medical care, long‐term services and supports, and address care quality. Recommendations present considerations for future research and opportunities to strengthen policies related to the care of persons living with AD/ADRD.

**Highlights:**

- Disparities related to AD/ADRD negatively impact diverse populations.
- Limited data on underrepresented groups make it difficult to assess the full scope of disparities.
- Increasing access to health information technology is necessary for reducing disparities.
- More information is needed to understand the impact of payment models on addressing disparities.

## 1 INTRODUCTION

Disparities related to Alzheimer's disease and Alzheimer's disease related dementias (AD/ADRD) are a priority concern for policymakers, practitioners, and researchers. Over the past several years despite a decline in AD/ADRD prevalence in the United States, disparities persist.^1^ These health disparities—defined as any health difference associated with social, economic, and environmental disadvantages^2^—adversely impact diverse populations across age, sex and gender, race and ethnicity, language, education, socioeconomic status, and place. For example, individuals from older age groups, women, Medicare–Medicaid dual‐eligibles, individuals living in rural areas, and older adults from racial and ethnic minoritized groups experience higher rates of AD/ADRD.^1^ Disparities are also often reflected in unequal access to timely diagnosis and anti‐dementia medications, as well as increased odds of hospitalizations and more aggressive life‐sustaining treatment at the end of life.^3^ Because many individuals living with AD/ADRD require assistance from family caregivers or paid direct care workers with routine daily activities, disparities often affect those who are part of their caregiving networks. This is evidenced by disparities in family caregiver emotional, financial, and physical burden, and limited access to supports for direct care workers such as AD/ADRD‐relevant training.^4^, ^5^ Health disparities threaten the well‐being of older adults living with AD/ADRD, contribute to intergenerational disadvantages, and challenge the sustainability of family caregivers and the health care workforce (e.g., clinicians; direct care workers). For these reasons, and given the growing and diversifying aging population, innovative strategies are needed to address AD/ADRD‐related health disparities.

Several factors shape the experiences of individuals that contribute to AD/ADRD‐related disparities. These factors include socioeconomic status, access to important health services, and cultural differences in health perceptions and practices.^6^ These factors also extend to contextual characteristics that encompass federal, state, and local policies, such as those related to service delivery and financing.^7^ However, many populations are not well represented in current research on AD/ADRD, making it difficult to understand the full scope of disparities for many older adults in the United States. In addition, the coronavirus disease 2019 (COVID‐19) global pandemic has accelerated shifts in health care's technological landscape (e.g., patient portal use, telehealth). However, accessibility has remained limited for racial and ethnic minoritized groups, as well as individuals living in poverty and/or in rural areas.^8^

In this perspective piece, we highlight research gaps and opportunities that policymakers, practitioners, and researchers should prioritize in order to meaningfully address AD/ADRD‐related disparities, based on recommendations discussed during *Session 4: Disparities in Health Care Access, Utilization, and Quality*, of the *2023 National Research Summit on Care, Services, and Supports for Persons Living with Dementia and Their Care Partners/Caregivers*.^9^ Specifically, we call attention to three areas for policy and research to tackle disparities related to the care of people living with AD/ADRD and their caregivers: (1) increased data availability and linkages across local, state, and federal levels; (2) health information technology use and related care access, quality, and costs; and (3) diverse health insurance models used to enable access to medical care and long‐term services and supports, and address care quality. Recommendations discussed in this perspective present pathways for future research as well as considerations for strengthening aging and disability policies related to the prevention, diagnosis, treatment, and support of older adults living with AD/ADRD.

## 2 STRATEGIES TO EXPAND DATA AVAILABILITY AND LINKAGES BETWEEN DATA SOURCES AT LOCAL, STATE, AND FEDERAL LEVELS

### 2.1 Identifying AD/ADRD populations

Much of what we know about disparities in care for those living with AD/ADRD is both advanced and limited by available data, possible data linkages, and data sharing among health care systems, community‐based organizations, researchers, or other working partners. A large body of literature regarding AD/ADRD has focused on the Medicare population. Although these studies use large samples for racial and ethnic groups, data from underrepresented groups and regions may be limited.^10^ For instance, researchers using the Master Beneficiary Summary File and the Chronic Conditions Warehouse algorithm to identify the incidence and prevalence of AD/ADRD among Medicare enrollees may not include Medicare Advantage (MA) enrollees, who represent over half of the entire Medicare population and are overrepresented by racial and ethnic minoritized groups.^11^, ^12^ Expanding diverse, representative data collection and linkages is essential for advancing science and policy. This includes leveraging existing measures of ADRD risk, burden, disparities, and resource use from large population‐based surveys and studies, such as the National Health and Aging Trends Study, Health and Retirement Study, and National Health Interview Survey. These sources complement administrative datasets such as Centers for Medicare & Medicaid Services (CMS) claims, which are prone to biases (e.g., selective diagnosis), offering critical insights into ADRD burden and care beyond administrative data. Identifying and implementing strategies to expand data collection, availability, and linkages, across various levels, is vitally important for advancing science and policy. It is also important to ensure that data enhancement strategies allow for the collection and sharing of quality data on race, ethnicity, and place‐based factors to increase representation of vulnerable and underrepresented populations. Such data infrastructure can facilitate deeper investigation of social determinants of health, enable examination of disparities in AD/ADRD care access and quality, and support a greater understanding of individual and structural intersectionality.

Too often, socially disadvantaged and minoritized populations and communities are underrepresented in research as a result of historic disinvestment in these marginalized groups. Although the U.S Department of Health and Human Services (HHS) and the CMS are taking steps toward improving administrative data reporting and sharing, future data collection and sharing efforts should focus on disaggregating Hispanic/Latino and Asian American populations, increasing data for Indigenous and Native American populations, and including data from U.S. territories such as Puerto Rico.^10^ In addition, for some of these groups and/or areas, the only data available regarding AD/ADRD are through the Mapping Medicare Disparities (MMD) (by Population) interactive tool.^13^ The MMD tool is publicly available and can identify disparities among Medicare subgroups regarding chronic condition prevalence, health care utilization, and outcome measures (e.g., ADRD prevalence, mortality, costs, hospitalization, readmissions, and emergency department visits).^13^ However, most of the indicators are only available for the fee‐for‐service population. As a result, missing data from these populations, and others like them, limit what we can learn and, ultimately, what can be done to improve AD/ADRD care.

### 2.2 Considerations for social determinants of health

In addition to being able to identify persons and populations impacted by AD/ADRD, recent efforts have enabled better collection and reporting of non‐medical factors affecting health, or social determinants of health. Although environmental conditions and sociodemographic information may still be limited in claims data, data such as the National Institute on Minority Health Disparities (NIMHD) PhenX toolkit and claims‐based Z‐codes make it possible to examine the role of social determinants of health.^14^, ^15^ The PhenX is a web‐based catalog of recommended measurement protocols (selected by experts) to include in studies with human participants. Z‐code claims for Medicare fee‐for‐service beneficiaries include problems related to education and literacy, unemployment, occupational exposure to risk factors, housing, social environment, upbringing (e.g., history of abuse in childhood, parent–child conflict), family, and psychosocial circumstances (e.g., imprisonment and other incarceration, exposure to disaster).^15^ In addition, with the 2024 Physician Fee Schedule Final Rule, CMS included payment for social determinants of health (housing insecurity, food insecurity, transportation needs, and utility difficulty) risk assessments provided during the annual wellness visit, behavioral visits, and evaluation and management visits including hospital discharge or transitional care.^16^

Given that health care organizations may already collect social determinants of health information in their electronic health records (EHRs), there are opportunities to create partnerships between local practitioners, organizations, and institutions to share data and improve access to care and services.^17^ However, careful consideration and intention are needed to ensure that minoritized groups are protected from unintended consequences (e.g., stigma, bias, lack of access to necessary resources) resulting from collecting data on social determinants of health in systems like EHRs.

### 2.3 Data interoperability and integration

In addition to obtaining more information about underrepresented populations and the environments in which they live, there is a crucial need to develop methods to improve data interoperability and integration or health information exchange across different health care settings and sources at various levels. This includes linking data from individual (e.g., sociodemographic, health, disability characteristics), local (e.g., aggregate measures of health disability and sociodemographic characteristics at the neighborhood‐level), state (e.g., availability of funding, states, and services for aging and AD/ADRD populations), and federal (e.g., changes to Medicare and Medicaid programs) levels across care settings outside health systems, such as nursing homes, adult day centers, or federally qualified health centers, as well as informal care settings. For example, when working to understand disparities it would be helpful to know if there is segregation within or across settings, how settings conceptualize equity and address disparities, and whether they offer dementia‐specific programming and supports. Similarly, there needs to be national standardization around the data that are collected and shared. There have been federal efforts to this end, including funding opportunities from the National Institute on Aging (NIA) to support researchers in developing innovative methods toward data harmonization across multiple sources and the Improving Medicare Post‐Acute Care Transformation Act of 2014 (IMPACT Act), which standardizes patient assessment data in post‐acute care settings.^18^, ^19^ This is important, given that few health care settings have standardized assessments that they use to report about their patients. For instance, nursing homes use the Minimum Data Set (MDS), and Medicare home health agencies use the Outcome and Assessment Information Set (OASIS). However, there is no standardized assessment tool for Medicaid Home and Community‐Based Services, making it difficult to understand care gaps and needs nationally, as well as to assess the quality of evidence produced.

### 2.4 Equitable data access

To ensure expanded data availability, data need to be made affordable and accessible for researchers across different settings. Health care data can be expensive and difficult to acquire and maintain for some institutions, particularly for those with limited resources, as well as for early career researchers who face market bottlenecks and competitive funding environments.^20^ These can create barriers for new investigators and scholars at different institutions and limit diversity and inclusion in research. Researchers should consider making their data available in institutional or public data libraries to promote transparency and to enable other researchers to build on existing findings and use data to support disparities research. Furthermore, efforts such as the NIA Data Linkage Program (LINKAGE) are needed to provide opportunities to improve data access to claims and other population‐based studies and sharing across different investigators and institutions.^21^ LINKAGE program researchers leverage this free resource to access data linked to CMS administrative claims as well as contribute their own datasets to be linked to claims. As of 2024, the LINKAGE program has 17 NIA‐funded studies that are currently linked to CMS data, including the Bluestone Physician Services, the Long‐term Effects of a Community‐based Volunteer Trial on Lifestyle Activity and Risk for Alzheimer's Disease; the Health and Retirement Study, the National Health & Aging Trends Study (NHATS), the Rush Alzheimer's Disease Center, and the Useful Field of View Training Randomized Control Trials. Including other population‐based cohorts and NIA‐funded datasets into the enclave could improve the diversity of study cohorts and research questions related to AD/ADRD.^22^ Given the existing challenges related to accurately identifying persons from racial and ethnic minoritized groups in administrative claims data,^10^ it is important that studies in the NIA LINKAGE program take steps to address this challenge. For instance, the NHATS oversamples Black older adults as well as those who are 85 years of age or older.^23^

## 3 EFFECTS OF HEALTH INFORMATION TECHNOLOGY ON CARE ACCESS, QUALITY, AND COSTS

### 3.1 Expanding health information technology in dementia care

In addition to identifying AD/ADRD populations and ensuring equitable access to data, the expansion of health information technology (HIT) has created opportunities and challenges for increasing access to and quality of health care services. HIT has the potential to transform health care delivery across diverse settings by facilitating multisector collaboration among health care providers, public health systems, and social services.^24^ Older adults living with AD/ADRD often have complex health needs that require coordination across health care systems, community services, and caregivers. Evidence suggests that HIT can enhance health care access, improve quality, reduce costs, and promote health equity,^24^, ^25^ and is in alignment with strategies such as the Alzheimer's Association's public health action plan to advance care and support for individuals with ADRD.^26^

HIT's role in improving care coordination and reducing disparities is critical for addressing fragmented care, which disproportionately affects AD/ADRD patients. HIT connects hospitals, outpatient services, long‐term care facilities, and community programs, improving continuity of care, minimizing redundancies, and significantly reducing readmissions and emergency department visits among older adults with ADRD.^27^, ^28^ HIT tools such as patient portals empower patients with features like prescription refill requests, access to clinical notes, and appointment scheduling, thereby enhancing their ability to manage their health.^29^ These tools have been shown to improve medication adherence and overall patient engagement.^30^, ^31^ In addition, HIT facilitates data sharing through health information exchanges that securely connect clinical patient data across hospitals, laboratories, and health care organizations, promoting coordinated care and reducing preventable emergency department visits for persons living with AD/ADRD through more accurate and efficient information sharing.^32^, ^33^

### 3.2 Addressing disparities in HIT adoption

Despite its potential, disparities in HIT adoption continue to pose significant barriers to achieving equitable health care delivery.^34^ Rural populations often face transportation barriers, thereby limiting their ability to access timely in‐person care.^35^ Studies have shown that virtual consultations facilitate earlier interventions for patients in rural areas, reducing health care delays and significantly improving access to timely care.^36^ However, rural residents were less likely to use such robust telehealth systems with advanced features. This disparity is driven primarily by limited broadband infrastructure in rural areas, leaving rural health care providers unable to implement or maintain advanced HIT systems.^37^

Similarly, older adults from racial and ethnic minoritized groups, who could significantly benefit from HIT infrastructure, are often less likely to access or use such systems due to social determinants of health, including limited digital literacy, broadband access, and economic constraints.^38^ Evidence suggests that a more equitably distributed HIT infrastructure is critical for providing access to these underserved populations. For example, studies have shown that Black patients who utilized HIT services experienced lower Medicare costs, highlighting the potential for HIT to improve care coordination and reduce health care expenditures in underserved communities.^39^

### 3.3 Advancing HIT adoption in long‐term care settings

Long‐term care (LTC) settings also face significant obstacles in HIT adoption.^40^, ^41^ Exclusion from the federal Meaningful Use program under the Health Information Technology for Economic and Clinical Health (HITECH) Act left LTC settings, such as skilled nursing facilities and adult day centers, lagging in EHR implementation. Many LTC facilities continue to rely on paper‐based systems or fragmented digital solutions, hindering interoperability with hospitals and other providers. For example, 92% of adult day centers lack interoperable health record systems, limiting their ability to manage medications, track health changes, and communicate with caregivers or clinical providers.^42^ Innovative HIT solutions like *CareMobi* offer a promising approach to addressing these challenges and strengthen supports for persons living with AD/ADRD, who often access services provided at adult day centers.^40^ *CareMobi* is a lightweight, low‐cost mobile application designed for adult day centers, enabling them to track medications, appointments, nutrition, and mood for their clients. The app allows caregivers to monitor these health indicators and share updates with family members and clinical providers, fostering better communication and care coordination. *CareMobi* helps identify emerging clinical issues early by providing real‐time insights into participants' health enabling timely interventions. Furthermore, because the app is cost‐effective and user‐friendly, it is accessible to under‐resourced centers that may lack the funding for more complex HIT solutions.

### 3.4 Policy advancements driving HIT integration

Recent policy advancements further bolster the development of HIT. The Guiding an Improved Dementia Experience (GUIDE) Model promotes team‐based care, fosters caregiver engagement, and delivers tailored services to meet the unique needs of dementia patients and their families.^43^ It may incentivize the adoption of HIT to improve care coordination among providers, family caregivers, and patients. Accountable care organizations (ACOs) incentivize HIT adoption by linking financial rewards to improved patient outcomes. Research demonstrates that combining telehealth services with ACO incentives significantly reduces Medicare costs for persons living with AD/ADRD, particularly in socially vulnerable areas and among racially and ethnically minoritized populations.^44^

Future efforts should focus on expanding broadband access, improving digital literacy, and incentivizing HIT adoption through value‐based payment models.^45^ Research should address the distinct technological needs of older adults and the infrastructure requirements of care settings, emphasizing culturally sensitive, user‐friendly designs that enhance telemedicine, virtual care, and medication management.

## 4 IMPACTS OF HEALTH CARE POLICIES AND POLICY MODELS ON CARE QUALITY AND ACCESS

Although the two largest public health insurance programs in the United States, Medicare and Medicaid, were both introduced in 1965, these are far from stagnant programs. Within Medicare, there has been a continuous shift away from traditional, fee‐for‐service Medicare, toward Medicare Advantage programs, which are run by private health insurance companies and face different financial incentives that try to encourage the provision of more value‐based health care. MA selection is an individual choice and thus raises concerns about selection and bias when evaluating these plans. MA plans have to cover all the benefits in traditional Medicare parts A and B but have a lot of flexibility in offering additional benefits and in setting their pricing schemes. This variation also means that there is variation in the quality of MA plans, as measured by a star rating system.

### 4.1 Medicare Advantage

As stated earlier, the growth in MA enrollment has been substantial, with 2023 being a notable year where over half of Medicare beneficiaries were enrolled in an MA plan. The growth in MA coverage has been greatest among Black and Hispanic older adults.^12^ It has been shown that these minoritized populations are also more likely to enroll in lower‐rated MA plans. Although this could be for a variety of reasons, the lack of access to high‐quality MA plans seems to be an important one. Park et al. found that high‐quality plans were less available to minoritized populations, and once access was accounted for, racial differences in enrollment in high‐quality MA plans almost disappeared.^46^

Enrollment in MA has implications for access to health care. MA plans often use a variety of measures to control utilization, including selectively contracting with providers willing to accept lower reimbursement rates, commonly referred to as their preferred provider network.^47^ These lower‐cost providers may come at the cost of high‐quality care; compared to traditional Medicare enrollees, MA beneficiaries are more likely to receive services from lower‐quality hospitals, home health agencies, and skilled nursing facilities.^47^ Specifically, Black and Hispanic patients with AD/ADRD are both more likely to enroll in MA plans and more likely to be admitted to lower‐resourced, lower‐quality, and segregated nursing homes.^48^

If someone is unsatisfied with their MA coverage, they can switch to traditional Medicare or select a different MA plan each year during open‐enrollment period. Indeed, disenrollment rates from MA have been increasing over time. Disenrollment rates are also much higher among individuals with diagnosed dementia than among those without dementia.^49^ Over a 5‐year period, almost half of MA enrollees (48%) who are not eligible for Medicaid have disenrolled from their initial MA contract.^50^ These 5‐year disenrollment rates are highest among Black and American Indian/Alaska Native enrollees. However, switching insurance, especially from MA to traditional Medicare, may be influenced by another insurance product, that is, the price and availability of Medigap plans, or insurance plans that are designed to help “fill the holes” in traditional Medicare by providing lower out‐of‐pocket costs or introducing out‐of‐pocket maximums. Medigap plan pricing and access is regulated at the state level. Park and Coe^51^ found indeed that these state‐level pricing regulations increased the price of Medigap plans, and thus lowered the enrollment rates in traditional Medicare from MA plans.

Another area of experimentation has been in care coordination, especially within MA plans. There are three types of Special Needs Plans (SNPs): Institutional SNPs (I‐SNPs) for people who need institutional‐level care; Chronic Care SNPs (C‐SNPs), for people with severe or disabling conditions; and Dual‐Eligible SNPs (D‐SNPs), for the population eligible for both Medicaid and Medicare. Enrollment in these SNPs, driven by D‐SNP enrollment, tripled between 2011 and 2021,^52^ and served disproportionate shares of Black and Hispanic beneficiaries. Few C‐SNPs focus on dementia care, making I‐SNP and D‐SNPs the more relevant plans for persons living with AD/ADRD.

Although these care management plans hold promise, in general, they are understudied due to difficulty in identifying enrollees in these plans, and the general difficulty with measuring health care utilization within MA plans until recently. I‐SNPs incentivize more care coordination through having one plan responsible for all health care spending, regardless of setting (nursing home vs outpatient vs hospital). Recent work has found that I‐SNPs can increase clinical management within the nursing home setting, decreasing costly and unnecessary hospital use.^40^ D‐SNPs have evolved into many different programs, which could better meet beneficiary needs, but also further complicate analysis and understanding the effectiveness of the plans.

### 4.2 Medicaid

Medicaid, the health insurance program for individuals with low incomes and/or disabilities, is the primary payer for long‐term services and supports in the United States, on which persons living with AD/ADRD rely heavily. Starting with the 1999 Supreme Court decision *Olmstead v. L.C*., Medicaid has been transforming to rid itself of an institutional bias in care delivery and has been rapidly growing its home and community‐based services (HCBS). Indeed, in 2013, Medicaid began to spend more on HCBS than on institutional care.^53^ However, states have a lot of flexibility in running their Medicaid programs, which leads to Medicaid being essentially 51 different insurance programs. That is especially true within the long‐term services and supports (LTSS) coverage and eligibility, where most of the benefits are considered optional and met through waiver programs. Given the lack of supports like national long‐term care insurance options, as well as state variation in eligibility and benefits, considerable differences and disparities exist in the availability and use of HCBS among persons living with AD/ADRD. Kim et al. found that more than 80% of dual‐eligible beneficiaries (individuals with both Medicare and Medicaid coverage) with ADRD received some form of Medicaid LTSS in 2016, but that ranged between 61% and 96% between states.^54^ The type of Medicaid LTSS used varies across states as well, ranging from less than 10% to more than 60% for home‐based LTSS.

In addition, there are considerable racial differences in HCBS utilization, both overall and for persons living with AD/ADRD. Yan and colleagues^55^ document that, among Medicaid HCBS users with dementia diagnosis, Black beneficiaries were less likely to use hospice and institutional care than their White counterparts between 2010 and 2013. However, Black beneficiaries were more likely to use medical equipment, personal care, transportation, home health, and other waiver services than their White counterparts. More needs to be done to determine if these disparities are driven by access barriers or personal preferences.

Although the use of LTSS has spread, our understanding of the value of these services, or even who is receiving such services, is quite limited. Many states have moved to managed care models within Medicaid and/or within LTSS, and there is widespread concern of the reliability of the data available on spending by service within many states that have adopted these payment models.^56^ The one article that estimates a causal relationship between HCBS use compared to nursing home use is based on data before 2012 and found that HCBS users were more likely to be hospitalized, across all races and for those with and without dementia.^57^ Since that work was done, Medicaid has changed their data reporting, complicating the ability to update those results. CMS, Assistant Secretary for Planning and Evaluation (ASPE), contractors, and researchers have been working to create new taxonomies to identify HCBS users and spending in the new data format, but considerable challenges remain.^57^

The fragmented U.S. insurance and care delivery system contributes to disparities in access, care quality, and costs of needed care and options to care for persons living with AD/ADRD in community settings. Some states have instituted new programs to better standardize and coordinate care and financing. For example, Washington State passed the Long‐Term Care Trust Act in 2019, creating the first state‐run long‐term care insurance benefit. Residents pay a modest tax on income during their working life into the WA Cares Fund, and upon disability onset (defined as needing help with three activities of daily living [ADLs]), residents are eligible for up to $36,500 benefits, indexed for inflation, to help offset the costs of the care.^58^ The first benefit is payable in 2025, so time will tell how successful it is in filling the financial gap as well as aging residents to age with dignity. New York, with the goal of integrating care and incentives, has moved to a fully‐managed care model for all dual‐eligible individuals over 21 years of age or older, needing more than 120 days of long‐term services and supports. Mandatory enrollment was achieved statewide in 2015. Research suggests that the move to managed care has increased personal care use^59^ and lowered nursing homes rates among individuals with dementia.^60^

### 4.3 Diagnostic and pharmaceutical advancements

As we turn the corner with the promise of new diagnostic pharmaceutical interventions that could delay dementia onset, we must also pay attention to the access to specialists, prescribing behavior of those physicians, and the availability of pharmaceutical drugs. Currently, the use of anti‐dementia drugs is more prevalent among White beneficiaries than among minoritized populations; female patients are more likely to receive medications for their ADRD than their male counterparts^61^; and AD patients in rural communities are more likely to be underserved in both education and supply of medication than their more urban counterparts.^62^ Part D, the prescription drug program within Medicare, is undergoing several changes that decrease out‐of‐pocket costs for beneficiaries, it remains to be studied if these changes in benefit design will help with these existing disparities in access and use, especially among the dual‐eligible population.

## 5 CONCLUSION

AD/ADRD‐related disparities remain a pressing issue that can only be addressed with intentional and innovative action. We present three areas of focus for researchers, policymakers, and practitioners to consider in the pursuit of health equity for persons living with AD/ADRD. First, there is a need for an expansion of available data and data linkages to acquire better knowledge about individuals at risk of and living with AD/ADRD. In addition, the ability to link data sources across local, state, and federal levels creates avenues by which interventions can better target factors such as social determinants of health (e.g., housing, transportation) and improve supports for persons living with AD/ADRD and their family caregivers. Second, recent years have seen an increase in the use of HIT in medical care and long‐term services and supports, but without addressing disparities in internet access and HIT utilization; existing gaps in care may widen and further negatively impact groups disparately affected by AD/ADRD. Third, health care policy and policy models are often responsible for financing medical care and long‐term services and supports received by persons living with AD/ADRD. More information is needed to understand the effectiveness of policies on quality‐of‐life outcomes for persons living with AD/ADRD. Recommendations highlight gaps and present opportunities for future research and aging and disability policies related to the care of persons living with AD/ADRD.

## CONFLICT OF INTEREST STATEMENT

The authors have no conflicts to disclose.

## CONSENT STATEMENT

No consent was needed for this work.Supporting Information

## Supporting information



Supporting information
